# Veterinary Medical Association, University of Pennsylvania

**Published:** 1898-04

**Authors:** M. Jacob

**Affiliations:** Secretary


### VETERINARY MEDICAL SOCIETY, UNIVERSITY OF PENNSYLVANIA.

MEETING was called to order at 8 o'clock P.M. Mr. H. Hoopes, who was appointed as a committee to see about the certificates, made a report at the last meeting. He said that the Society bought sixty certificates for seventy-five dollars at Avil & Co.'s. Thirty were taken last year, and the remainder are to be taken this year.

It was moved and seconded that the Executive Committee be instructed to buy the other certificates of Avil & Co., and have enough stamped for the members of the Classes of 1899 and 1900.

Mr. E. Newcomer made a report, and said that he had the library magazines bound.

Mr. Thos. Sharpless, of Chester County gave a very interesting talk on the "Breeding and Care of Swine." It was a very instructive talk, and Mr. Sharpless demonstrated how well he understands this part of animal husbandry. He is one of the most extensive breeders of swine in this State; his favorites are the Chester White swine.

Mr. Walters, of Chester County, accompanied Mr. Sharpless.

Mr. S. McClure, of the Class of '98, was very active and earnest in getting up the interesting programme for the last meeting, and the Society extended him a vote of thanks.

M. JACOB,
Secretary.

